# Physiognomy

**Published:** 1856-02

**Authors:** 


### PHYSIOGNOMY.

Is there anything in it? No. 604 of LITTEL'S LIVING AGE, of which John Quincy Adams wrote ten years ago, "Of all the Periodical Journals devoted to literature and science which abound in Europe, and in this country, this appears to me the most useful"—contains an unpublished letter of Bishop Bonner, whose rubicund, fat, comely, jolly looking presence—whose smooth, round, florid, pleasant looking countenance occasioned so many jokes among his cotemporaries. This letter, the authenticity of which is undoubted, gives additional ground for believing that the world's judgment of Bonner's character was just, as being one of the most cold-hearted, malignant, murderous wretches that ever disgraced humanity. Here is a face all sunshine and a heart all black as night. Then again, there is a like contradiction in the faces of those who, too precise and dignified to ever laugh outright, wear an everlasting simmer; the stereotype smile, beautiful as an icicle—and as cold, covering a temper, which for all that is treacherous, unfeeling and vindictive, has no precedence except among archangels fallen. The hearty, free-souled, loud guffaw, we are never afraid of; but we do instinctively shrink as from an adder's fang, from that soft, that ready, that snakey smile.

